# 644. Phenotypic and Genomic Analysis of Novel, Fastidious, Gram-negative Bacilli Isolated from Clinical Wound Specimens

**DOI:** 10.1093/ofid/ofab466.841

**Published:** 2021-12-04

**Authors:** Benjamin Liu, Keith Simmon, Mark Fisher

**Affiliations:** 1 University of Utah School of Medicine/ARUP, Salt Lake City, Utah; 2 ARUP, Salt Lake City, Utah; 3 ARUP Infectious Diseases Laboratory, Salt Lake City, UT

## Abstract

**Background:**

Animal bites are considered the thirteenth leading cause of nonfatal ED visits. Epidemiology studies have shown a rise in dog bites during the COVID-19 pandemic in the U.S. In Oct. 2020, we received a facultatively anaerobic, non-hemolytic Gram-negative rod (OL1) from a dog bite wound for identification. 16S rRNA gene sequencing showed OL1 was 95.9% identical to *Ottowia pentelensis* in the family *Comamonadaceae*. Our historical sequence database revealed 8 additional isolates (OL2-OL9) from hand wounds/abscesses (including 3 dog bites) since 2012 that had ⩾ 99.8% identity with OL1. Most other *Ottowia* sp. have been isolated from industrial and food sources, with no reports from patient samples. As these clinical isolates likely represent a novel *Ottowia* species, we aimed to characterize them using both phenotypic and genomic approaches.

**Methods:**

The OL isolates were tested in API 20 NE panels (8 conventional and 12 assimilation tests) for 4 d. Paired-end genomic DNA libraries (Nextera DNA Flex Library Prep, Illumina) were sequenced as 150 nt reads by Illumina NovaSeq. *De novo* assembly, annotation, functional prediction, and phylogenetic analyses were performed with Geneious, PATRIC, and web-prediction databases. Strain comparison was done with StrainTypeMer.

**Results:**

All 9 OL isolates were negative for indole, urea, arginine, esculin, PNPG, glucose fermentation and carbohydrate assimilation tests. Potassium gluconate assimilation and gelatin hydrolysis were positive for 5 and 4 isolates, respectively. StrainTypeMer showed the isolates from different patients were not closely related, but 2 from the same patient were indistinguishable. The estimated genome size was ~3.1 Mbp, with 66.1% G/C, and ~3523 coding genes. Potential virulence factors (BrkB and MviM), multidrug efflux systems (MdtABC-TolC and Bcr/CflA), and 1-2 intact prophages were identified. Genomic phylogenetic analysis with RAxML showed the OL isolates clustered separately from all known *Ottowia* spp.

**Conclusion:**

These OL isolates are fastidious, Gram-negative bacilli from clinical wound specimens, and are associated with dog bites. Genomic and 16S rRNA gene sequence analysis suggests these isolates constitute a novel species within the family *Comamonadaceae*.

**Disclosures:**

**All Authors**: No reported disclosures

